# Multiple Calcaneus Secundarius Ossicles Presenting with Anterior Foot Pain: A Case Report Highlighting Characteristic Imaging Features

**DOI:** 10.3390/jcm15083122

**Published:** 2026-04-20

**Authors:** Ki Jin Jung, Eui Dong Yeo, Jeong Han Nam, Woo Jong Kim

**Affiliations:** 1 Department of Orthopaedic Surgery, Soonchunhyang University Hospital Cheonan, 31, Suncheonhyang 6-gil, Dongam-gu, Cheonan 31151, Republic of Korea; c89546@schmc.ac.kr (K.J.J.); 137996@schmc.ac.kr (J.H.N.); 2 Department of Orthopaedic Surgery, Veterans Health Service Medical Center, Seoul 05368, Republic of Korea; angeldoctor@bohun.or.kr

**Keywords:** calcaneus secundarius, accessory ossicle, bone marrow edema, diagnostic pitfall, fibrous connection

## Abstract

**Background**: Calcaneus secundarius (CS) is an accessory ossicle located at the anterior aspect of the calcaneus and is typically an incidental and asymptomatic radiographic finding. However, it may become symptomatic following trauma or repetitive mechanical stress and can mimic anterior calcaneal process fracture or tarsal coalition, leading to diagnostic confusion. The presence of multiple independent CS ossicles represents a rare morphological variant and a potential source of diagnostic ambiguity. **Methods**: We report the case of a 19-year-old male soldier who presented with progressive anterior foot pain following soccer activity without a clearly identifiable traumatic event. Radiographs, computed tomography (CT), and magnetic resonance imaging (MRI) were performed to evaluate the underlying pathology. **Results**: CT demonstrated two separate, well-corticated accessory ossicles adjacent to the anterior calcaneal process without bony continuity. MRI revealed focal bone marrow edema (BME) at the calcaneus–ossicle interface, suggesting mechanical irritation at the fibrous connection. Due to persistent symptoms and concordant imaging findings, surgical excision was performed, resulting in immediate pain relief and return to full daily and sports activities without recurrence at the 1-year follow-up. **Conclusions**: Multiple CS ossicles may produce fragment-like imaging appearances and increase the risk of misdiagnosis. Recognition of characteristic imaging features, particularly well-corticated ossicles and focal BME at the ossicle–calcaneus interface, together with clinical correlation, is essential for accurate diagnosis and appropriate management in patients with persistent anterior foot pain.

## 1. Introduction

Calcaneus secundarius (CS) is an accessory ossicle located adjacent to the anterior process of the calcaneus and the calcaneocuboid joint, most commonly reported as an incidental asymptomatic finding on radiographic examination [1,2,3]. First described by Stieda in 1869 [2], CS is a rare anatomical variant with a reported prevalence of approximately 3.4% in skeletal studies [1]. Although typically asymptomatic, CS may become clinically relevant following trauma or repetitive loading, particularly when the fibrous or cartilaginous interface between the ossicle and the calcaneus is disrupted or stressed [2,4,5]. The interface between the ossicle and the calcaneus may represent a fibrous connection, which can serve as a potential source of localized pain under mechanical loading [2,4,6].

When symptomatic, CS can present with imaging features that closely resemble those of an anterior process fracture of the calcaneus or calcaneonavicular coalition, posing significant diagnostic challenges [6,7,8,9]. Such resemblance may lead to misdiagnosis in patients presenting with midfoot pain following minor trauma, potentially resulting in inappropriate management, including unnecessary surgical intervention or delayed treatment. Although CS is increasingly recognized as a potential source of midfoot pain, no definitive diagnostic criteria have been established, and diagnosis remains largely dependent on the integration of clinical and imaging findings [3,9]. Computed tomography (CT) is particularly useful for confirming the absence of bony union between the ossicle and the calcaneus. Magnetic resonance imaging (MRI), particularly fat-suppressed T2-weighted sequences, is effective in identifying bone marrow edema at the CS–calcaneus synchondrosis, whereas T1-weighted images are useful for anatomical delineation [5,6,10]. Importantly, bone marrow edema is not exclusive to CS; it has also been documented in fibrous tarsal coalition, further complicating the differential diagnosis [6,10,11]. The key imaging and clinical features relevant to this differential diagnosis are summarized in Table 1.

While CS has been described predominantly as a single ossicle in the existing literature [2,4,5], the presence of multiple independent ossicles represents an exceptionally rare morphological variant. To the best of our knowledge, cases of multiple calcaneus secundarius ossicles have been rarely reported in the literature, making this a unique morphological variant with distinct diagnostic and therapeutic implications [4,5]. Such a presentation may further increase the likelihood of misinterpretation as post-traumatic pathology due to its resemblance to fracture fragments [7,8,9]. However, the clinical and imaging characteristics of multiple CS ossicles, as well as their implications for diagnosis and management, remain poorly defined.

We herein report a case of multiple CS ossicles presenting with forefoot pain. This case aims to present characteristic imaging findings across plain radiography, CT, and MRI, highlight the diagnostic pitfalls associated with this rare morphological variant, and support improved diagnostic accuracy and appropriate clinical decision-making.

## 2. Case Presentation

This case report was approved by the Institutional Review Board (IRB) of Soon-chunhyang University Cheonan Hospital, Cheonan, South Korea (IRB No. 2026-02-008). The patient provided written informed consent for the publication of this report and the accompanying images.

A 19-year-old male soldier presented with a 3-month history of progressive left anterior foot pain. The pain was aggravated by prolonged ambulation and physical activity, which significantly impaired his military duties. He denied any history of acute trauma or prior foot surgery. His initial pain intensity was rated 8 on the Visual Analog Scale (VAS), and his functional status was assessed using the American Orthopaedic Foot and Ankle Society (AOFAS) score, which was 64 at presentation.

Physical examination revealed localized tenderness over the anterior process of the calcaneus. The patient reported pain with ambulation. No swelling, deformity, or neurovascular deficits were noted. Range of motion of the ankle and subtalar joints was within normal limits.

**Figure 1:**
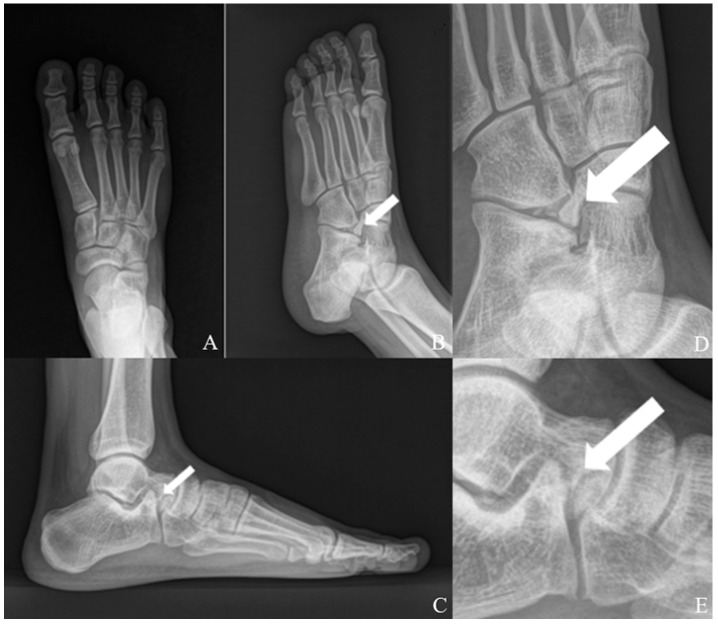
Preoperative plain radiographs of the left foot. (**A**) Anteroposterior, (**B**) oblique, and (**C**) lateral views demonstrate an accessory ossicle adjacent to the anterior process of the calcaneus (arrow). (**D**,**E**) Magnified views show a well-defined, smoothly marginated ossicle without features suggestive of an acute fracture fragment.

**Figure 2:**
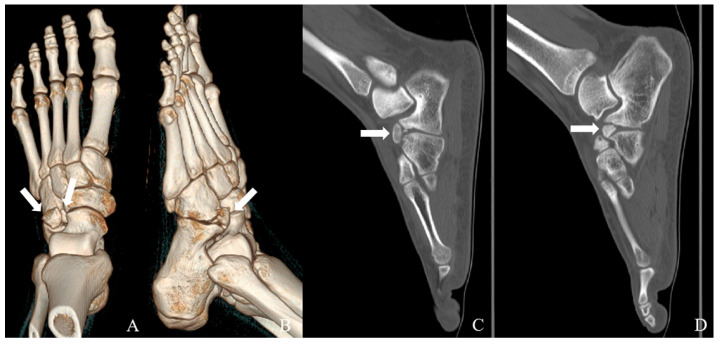
Computed tomography images of the left foot demonstrating multiple calcaneus secundarius ossicles. (**A**,**B**) Three-dimensional reconstructed images show two separate, well-corticated accessory ossicles adjacent to the anterior process of the calcaneus (arrows). (**C**,**D**) Sagittal CT images demonstrate that both ossicles have smooth cortical margins without bony continuity with the calcaneus, confirming the absence of osseous coalition.

**Figure 3:**
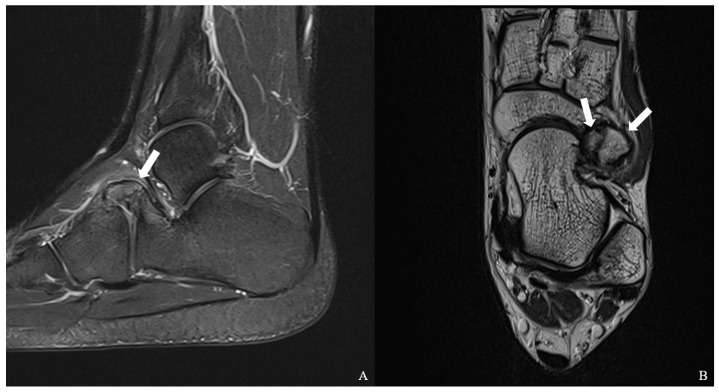
Magnetic resonance imaging of the left foot. (**A**) Fat-suppressed sagittal T2-weighted image demonstrates focal high signal intensity consistent with bone marrow edema at the calcaneus–ossicle interface (arrow), without a definite fracture line. (**B**) Coronal T1-weighted image demonstrates two well-defined accessory ossicles adjacent to the anterior process of the calcaneus (arrows), showing smooth cortical margins without articular continuity.

Plain radiographs of the left foot demonstrated an accessory ossicle anterior to the calcaneal anterior process with smooth, well-corticated margins, without features suggestive of an acute fracture (Figure 1). Although plain radiographs suggested a single accessory ossicle, further evaluation with CT revealed the presence of two distinct ossicles. Computed tomography (CT) confirmed the presence of two distinct ossicles anterior to the calcaneal anterior process, with no evidence of bony union with the calcaneus (Figure 2). Magnetic resonance imaging (MRI) revealed bone marrow edema at the calcaneus-ossicle interface on fat-suppressed T2-weighted sequences, consistent with mechanical irritation at the fibrous connection site (Figure 3).

The patient was initially managed conservatively with nonsteroidal anti-inflammatory drugs (NSAIDs) for 2 months; however, symptoms persisted without improvement, leading to the decision for surgical intervention.

**Figure 4:**
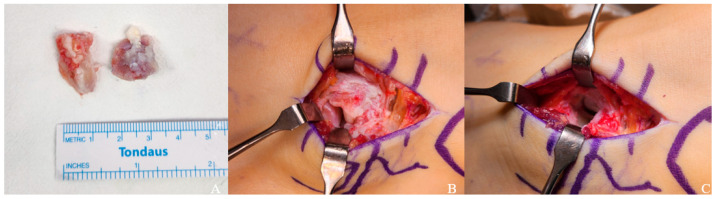
Intraoperative and gross findings of the excised calcaneus secundarius ossicles. (**A**) Gross photograph of the excised specimens showing two separate ossicles with smooth cortical surfaces. (**B**) Intraoperative view demonstrating the accessory ossicles located anterior to the calcaneal anterior process, connected to the calcaneus by fibrous tissue. (**C**) Intraoperative view after complete excision of the ossicles, showing the surgical bed without residual ossicle. The excised ossicles demonstrated smooth articular cartilage surfaces as well as irregular areas of fibrous tissue attachment. No definite true synovial articulation with adjacent tarsal bones was identified.

**Figure 5:**
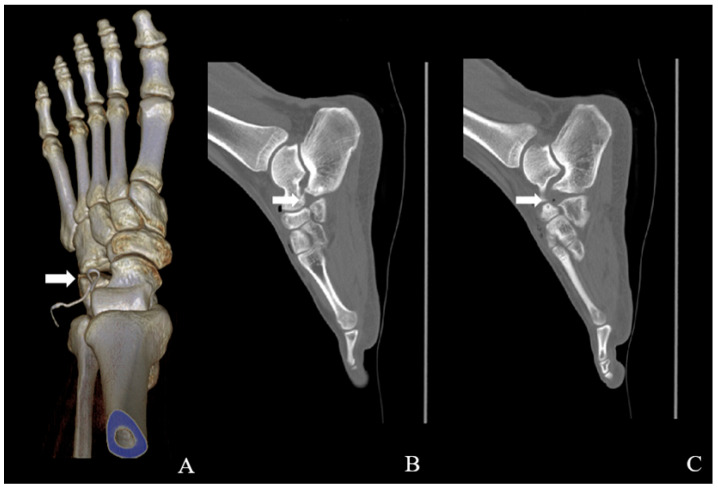
Postoperative computed tomography images of the left foot. (**A**) Three-dimensional reconstructed image demonstrates the calcaneus following surgical excision of the accessory ossicles (arrow). (**B**,**C**) Sagittal CT images show complete removal of the previously identified ossicles, with no residual ossicle or bony fragment at the anterior process of the calcaneus (arrow).

Surgical excision was performed in April 2024. Intraoperatively, two distinct ossicles were identified anterior to the calcaneal anterior process (Figure 4). The ossicles were connected to the calcaneus via fibrous tissue, with no bony union identified. The two ossicles were also interconnected by fibrous tissue. The ossicles appeared to be in contact with adjacent structures, including the cuboid, without clear evidence of a true synovial articulation. The surfaces of both the ossicles and the adjacent structures were smooth and showed no degenerative changes. These findings were consistent with a fibrous connection rather than a true articulation. Both ossicles were completely excised. Gross examination of the excised specimens demonstrated smooth articular cartilage surfaces without degenerative changes, as well as irregular areas of fibrous tissue attachment. The articular surfaces were considered to be related to the calcaneus, although no definite true synovial articulation was identified (Figure 4). Postoperative CT confirmed complete removal of the previously identified ossicles, with no residual ossicle or bony fragment at the anterior process of the calcaneus (Figure 5).

Histopathological analysis confirmed two pieces of bony tissue with an aggregate size of 2.0 × 1.5 × 0.5 cm, consisting of bone, cartilage, and fibrous tissue. The preservation of normal articular cartilage suggested that the patient’s pain was attributable to mechanical irritation at the fibrous connection interface rather than articular cartilage degradation.

At the 1-year follow-up, the patient reported near-complete resolution of pain, with a VAS score of 1 and an AOFAS score of 90. No postoperative complications or recurrence was observed. The patient reported that he was able to return to normal activities without limitation and was satisfied with the outcome.

## 3. Discussion

CS is an uncommon accessory ossicle located at the anterior aspect of the calcaneus, adjacent to the anterior calcaneal process and the calcaneonavicular joint [2,3]. It arises from an incompletely fused secondary ossification center and is most often connected to the calcaneus by a synchondrosis or fibrous tissue rather than a true articulation [2,4]. Calcaneus secundarius has been described as a well-corticated ossicle with smooth margins, typically asymptomatic and identified incidentally on imaging. Recognition of its characteristic location and morphology is important to avoid misinterpretation as a fracture fragment or tarsal coalition [2,4].

Although CS is generally regarded as an asymptomatic incidental finding, it may become a clinically relevant source of anterior foot pain when the fibrous or synchondral connection to the calcaneus is subjected to repetitive mechanical stress [2,4,5]. In the present case, no acute traumatic event was identified; rather, symptoms developed insidiously following soccer activity, suggesting that repetitive mechanical loading at the fibrous connection was the likely precipitating factor. This mechanistic distinction is clinically important, as it implies that symptomatic CS may arise not only in the context of acute trauma but also through cumulative mechanical irritation in physically active individuals.

The majority of previously reported symptomatic CS cases have described a single ossicle, and the occurrence of two morphologically distinct ossicles represents a particularly rare variant [2,4,5]. In the present case, CT identified two independent ossicles: an ovoid ossicle located adjacent to the calcaneocuboid joint and a triangular ossicle adjacent to the talonavicular joint, both situated anterior to the calcaneal anterior process. Each ossicle demonstrated well-corticated margins without bony continuity, consistent with previously described imaging features of the calcaneus secundarius [2,4,9]. The presence of multiple ossicles may amplify diagnostic uncertainty, as overlapping fragments can obscure the characteristic morphology of CS and simulate post-traumatic pathology or tarsal coalition on plain radiographs [7,8,9].

Multimodality imaging plays a critical role in the diagnosis of symptomatic CS. CT is particularly valuable in delineating ossicle morphology, cortical integrity, and spatial relationships to adjacent structures, thereby distinguishing CS from acute fracture or calcaneonavicular coalition [3,9]. In contrast, MRI provides functional information by identifying bone marrow edema (BME) and associated soft tissue changes. In the present case, MRI demonstrated focal BME at the calcaneus–ossicle interface, rather than diffusely involving the ossicle, suggesting localized mechanical irritation at the fibrous connection site. This finding is clinically relevant, as it suggests that the pain likely originates from micromotion at the fibrous interface rather than from intrinsic pathology of the ossicle itself. Such a pattern of BME may therefore serve as an important imaging feature in identifying the pain-generating site and differentiating symptomatic CS from an asymptomatic accessory ossicle [5,6].

It is important to distinguish CS from tarsal coalition, as these entities are conceptually different yet may demonstrate overlapping imaging features. CS represents an accessory ossicle arising from an unfused secondary ossification center, whereas tarsal coalition is defined as an abnormal connection between adjacent tarsal bones [6,10]. However, in certain cases, CS may be connected to the calcaneus by fibrous or cartilaginous tissue, resulting in a functional interface that can mimic a fibrous coalition on imaging. In such situations, the distinction between an accessory ossicle with a fibrous connection and a true fibrous coalition may be ambiguous, and diagnosis should be based on a comprehensive assessment of ossicle morphology, anatomical location, clinical context, and associated imaging findings. Misinterpretation of this entity may therefore lead to unnecessary surgical intervention or inappropriate management.

There are no established diagnostic criteria for CS, and diagnosis relies on a comprehensive integration of clinical and imaging findings [2,4]. In asymptomatic cases, CS is characterized by a smoothly margined, well-corticated ossicle at the anterior calcaneus without reactive changes [2,3]. In symptomatic cases, the additional presence of BME at the calcaneus-ossicle interface on MRI, corresponding to the site of maximal clinical tenderness, supports the diagnosis of symptomatic CS with mechanical irritation at the fibrous connection [5,6,11]. CT and MRI should therefore be regarded as complementary rather than interchangeable modalities: CT establishes the morphological diagnosis, while MRI identifies the pain-generating site and guides treatment planning.

Conservative management, including activity modification, anti-inflammatory medication, and orthotic support, should be considered the initial treatment strategy [2,4]. In the present case, two months of conservative treatment with NSAIDs failed to provide adequate symptom relief, and surgical excision was subsequently performed. Intraoperative findings confirmed two ossicles connected to the calcaneus and to each other via fibrous tissue, without degenerative changes in the adjacent articular cartilage. Histopathological examination demonstrated bone, cartilage, and fibrous tissue, consistent with a fibrous connection between the ossicles and the calcaneus. The patient achieved near-complete symptom resolution at one-year follow-up, with VAS improving from 8 to 1 and AOFAS score from 64 to 90, demonstrating that surgical excision provides reliable and durable symptom relief in appropriately selected patients with multiple CS ossicles refractory to conservative treatment [12,13].

This case underscores the importance of including CS-related pathology in the differential diagnosis of midfoot pain in physically active individuals, even in the absence of a discrete traumatic event [2,7,8,9,10,14]. When multiple ossicles or fragment-like appearances are encountered on imaging, clinicians should consider the possibility of a multiple CS variant with mechanical irritation at the fibrous interface, rather than prematurely attributing findings to fracture or tarsal coalition [6,7,8,9,10]. Increased awareness of this entity, combined with systematic multimodality imaging assessment, may reduce diagnostic delay and facilitate timely and appropriate management.

## 4. Conclusions

CS is an uncommon anatomical variant that should be included in the differential diagnosis of persistent midfoot pain, particularly in physically active individuals engaging in repetitive loading activities. This case highlights an exceptionally rare presentation of multiple morphologically distinct ossicles, which may closely mimic post-traumatic fracture fragments or tarsal coalition on plain radiography, thereby increasing the risk of diagnostic confusion and potential mismanagement. Accurate diagnosis requires careful integration of clinical findings with multimodality imaging: CT provides detailed morphological assessment of ossicle cortication, number, and spatial relationships, while MRI identifies the pain-generating site through detection of bone marrow edema at the fibrous interface. It is important to recognize that CS with a fibrous tissue connection to the calcaneus may functionally mimic a fibrous coalition on imaging; however, these entities remain conceptually distinct, and diagnosis should not be made on imaging alone without consideration of clinical context and ossicle morphology. Recognition of these characteristic imaging features is essential to avoid misinterpretation as fracture or coalition and to prevent unnecessary or inappropriate intervention. In patients with persistent symptoms refractory to conservative treatment, surgical excision of the ossicle can provide reliable pain relief and functional recovery, as demonstrated by the favorable outcomes observed at one-year follow-up in the present case.

## Figures and Tables

**Table 1:** Key clinical and imaging features for differential diagnosis of calcaneus secundarius.

Feature	Calcaneus Secundarius	Anterior Calcaneal Process Fracture	Calcaneonavicular Coalition
Typical history	Minor trauma or overuse	Acute trauma	Chronic, often non-traumatic
Cortical margin	Smooth and well-corticated	Irregular	Variable
Articular continuity	Absent (fibrous interface)	Disrupted	Present
MRI bone marrow edema	Confined to synchondrosis	Diffuse	Present in fibrous type [10]
Diagnostic implication	Accessory ossicle (may be symptomatic)	Acute fracture	Congenital coalition

## Data Availability

All data generated or analyzed during this study are included in this published article.

## Abbreviations

The following abbreviations are used in this manuscript:

CS	Calcaneus secundarius
CT	Computed tomography
MRI	Magnetic resonance imaging
AOFAS	American Orthopaedic Foot and Ankle Society
BME	Bone marrow edema
